# Bioderived Pickering Emulsion Based on Chitosan/Trialkyl Phosphine Oxides Applied to Selective Recovery of Rare Earth Elements

**DOI:** 10.1021/acsami.3c10233

**Published:** 2023-12-13

**Authors:** Byron Lapo, Sandra Pavón, Javier Hoyo, Agustín Fortuny, Paul Scapan, Martin Bertau, Ana María Sastre

**Affiliations:** † Department of Chemical Engineering, Universitat Politècnica de Catalunya, EPSEVG, Av. Víctor Balaguer 01, 08800 Vilanova i la Geltrú, Spain; ‡ School of Chemical Engineering, Technical University of Machala, UACQS, BIOeng, 070151 Machala, Ecuador; § Institute of Chemical Technology, TU Bergakademie Freiberg, Leipziger Straße 29, Freiberg 09599, Germany; ∥ Department of Chemical Engineering, Universitat Politècnica de Catalunya, ETSEIB, Diagonal 647, 08028 Barcelona, Spain; ⊥ Department of Physical-Chemistry, Universitat de Barcelona, 08028 Barcelona, Spain; # Fraunhofer Institute for Ceramic Technologies and Systems IKTS; Fraunhofer Technology Center for High-Performance Materials THM, Am St.-Niclas-Schacht 13, 09599 Freiberg, Germany

**Keywords:** yttrium, Cyanex 923, solvent extraction, adsorption, urban mining

## Abstract

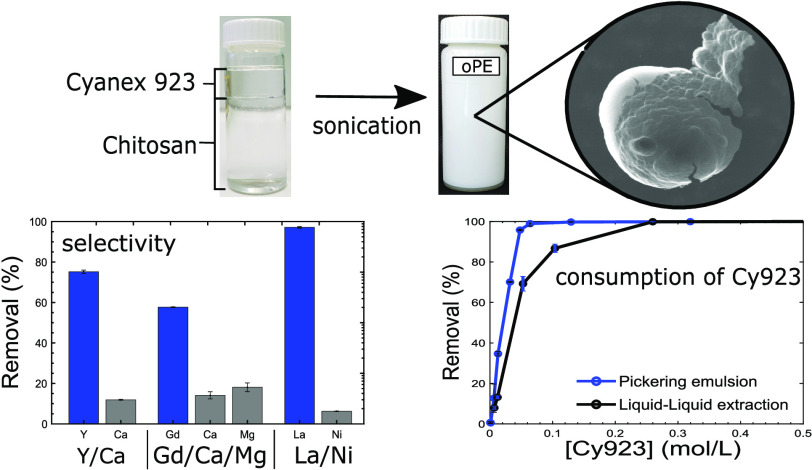

A novel biobased pickering emulsion (PE) material was prepared by the encapsulation of Cyanex 923 (Cy923) into chitosan (CS) to selectively recover rare earth elements (REEs) from the aqueous phase. The preparation of PE was optimized through sequentially applying a 2^3^ full factorial design, followed by a 3^3^ Box–Behnken design varying the Cy923 content, CS concentration, and pH of CS. The material was characterized by Fourier transform infrared (FTIR) spectroscopy, scanning electron microscopy (SEM), optical microscopy, rheological, compositional, and stability measurements. The resultant material was evaluated in the removal of yttrium by pH influence, nitrate concentration, kinetics, equilibrium isotherms, reusability, and a comparison with liquid–liquid (L–L) extraction and tested in a real scenario to extract Y from a fluorescent lamp powder waste. In addition, the selectivity of PE for REE was investigated with Y/Ca, Gd/Ca, and La/Ni systems. PE extracts REE at 1 ≤ pH ≤ 5 at nitrate concentrations up to 2 mol/L. The kinetics and equilibrium studies showed reaction times <5 min and a maximum sorption capacity of 89.98 mg/g. Compared with L–L extraction, PE consumed 48% less Cy923 without using organic diluents. PE showed a remarkable selectivity for REE in the systems evaluated, showing separation factors of 22.62, 9.35, and 504.64 for the blends Y/Ca, Gd/Ca/Mg, and La/Ni, respectively. PE showed excellent selectivity extracting Y from a real aqueous liquor from the fluorescent lamp powder. PE demonstrates to be an effective and sustainable alternative for REE recovering due to its excellent efficiency in harsh conditions, favorable green chemistry metrics, and use of a biopolymer material in its composition avoiding the use of organic solvents used in L–L extraction.

## 1 Introduction

Rare earth elements (REEs) are extensively used in modern technological devices including catalysts, lighting, magnets, batteries, advanced electronics, and military applications.^1^ The high demand for these valuable metals required the production of 280 000 metric tons of REE oxides in 2021, and a higher demand for the upcoming decades is expected.^2^ 76% of the natural reserves of REE are located in a bunch of countries (China, Brazil, and Russia),^3^ which represents price volatility as well as uncertainty in future availability for those countries without REE reserves. Consequently, the development of “urban mining” will simultaneously reduce the geolocation dependence and the health and environmental threat that represents the presence of REE in water resources.^4^ The recovery of these valuable metals has been mainly conducted by hydro- or pyro-metallurgical approaches. Hydrometallurgical processes include different techniques such as solvent extraction, ion exchange, precipitation, electrochemical processes, adsorption, and/or their related technologies.^5,6^

Among the cited technologies, liquid–liquid (L–L) solvent extraction and ion exchange are mostly applied on an industrial level. In both technologies, extractant molecules (EMs) have been extensively investigated with respect to chemoselectivity, efficiency, and stability toward REE recovery.^7^ However, in L–L extraction, concentrations of up to 90% of organic diluents such as kerosene or dodecane must be used to reduce the viscosity allowing efficient contact between EM and the metal to be extracted, but negatively contributing to hazardous chemicals input, increasing the costs and environmental footprint.^8^ On the other hand, in liquid–solid REE recovery such as ion exchange and adsorption, EM have been incorporated into the sorbent material to enhance the selectivity by blending and/or impregnation procedures.^9^ However, those procedures resulted in low REE uptake capacities (<50 mg/g), long reaction times (hours), and, in particular for resins, low recovery ratios (<50%). Impregnation induces weak binding between EM and sorbent materials providing unstable functionality coupled to the larger size (lower ratio contact area/volume) of its capsules (micromillimeter size). In addition, various green materials based on an ion imprinted technique such as La(III)IP-CS/PVP^10^ and straw-supported ion imprinted polymer^11^ have been tested with good performance for La selective removal, and materials applied to solid phase extraction (SPE) developed to enrich the concentration of REE prior its quantitative analysis. For example, poly(pyrrole-1-carboxylic acid),^12^ poly(caffeic acid),^13^ or oxidized graphene oxide^14^ demonstrated excellent selectivity toward REE but limited sorption capacity under 12 mg/g. Therefore, further research on green approaches to extract REE is required for avoiding the use of harsh chemicals and reducing EM and energy consumption.

Pickering emulsions (PEs) are presented as a sustainable approach for REE recovery. PEs are nano- or microsized emulsions stabilized by solid particles, which presently are gaining considerable interest in food, cosmetic, pharmaceutical, catalytic, and environmental fields.^15^ PE is formed by the incorporation of a dispersed phase (e.g., hydrophobic compound) into a continuous phase (e.g., wetted pickering particles),^16^ resulting in materials with enhanced resistance to destabilization phenomena.^17^ PE is usually obtained by several methods, such as ultrasound cavitation, high-pressure homogenizers, and microfluidizers. The simplicity, fast operation, and low energy consumption render ultrasound emulsification, the greener method.^18^ PEs have been recently used for recovering copper^19^ and cadmium^20^ from the aqueous phase probing the capability of PEs for metal removal from the aqueous phase.

PEs can be designed to perform selective REE recoveries in the aqueous phase by the incorporation of selective extractant molecules (EMs) into a wetted renewable biopolymer using high-frequency ultrasound. The oil-like nature of the EM (e.g., phosphine oxides) acts as a disperse phase, while a biopolymer (e.g., chitosan) is used as a structural compound for solid pickering particles, obtaining a selective and bioderived extractant material. Cyanex 923 (Cy923) is a suitable EM to be incorporated in PE that can be used in extended pH ranges under mild acid conditions.^21^ This molecule has been successfully applied for recovering REE from aqueous phase by solvent extraction^22^ and membrane technology.^23^ On the other hand, chitosan (CS) is an excellent renewable material candidate to serve as pickering particles. CS is considered a nonadsorbing polysaccharide that combines the electrosteric and viscosifying stabilization mechanisms, forming a rigid elastic network in the aqueous phase and consequently improving the stability of PE.^24^

The application of PE in metal recovery anticipates several advantages, including (i) the possibility of performing selective recoveries,^25^ (ii) absence of organic diluents (e.g., kerosene, dodecane),^26^ and (iii) possibility of using sustainable materials as pickering particles. These advantages contribute to the sustainability of the process in terms of reducing costs and environmental impact.

The current work is focused on the development and evaluation of PE as an alternative biosourced material for selective REE recovery. The optimal conditions for PE manufacture were determined following a design of experiments (DoE), and the optimal product was characterized and assessed in Y recovery. Yttrium (Y) was considered due to its huge importance in the REE market particularly related to lighting applications.^27^ A simple strategy of combining the selective properties of an EM with the capability of CS to form PE by a one-step ultrasonic emulsification was carried out. This route permits the reduction of Cy923 consumption and avoids the use of toxic solvents such as kerosene, typically used in L–L processes, which in turn contributes to the green engineering of REE recovery.

## 2 Materials and Methods

### 2.1 Chemicals

Cy923, composed by 4 trialkyl phosphine oxides including, dioctylhexyl-phosphine oxide (37.4%), octyldihexyl-phosphine oxide (30.4%), trioctyl-phosphine oxide (16%), and dihexyl-phosphine oxide (8.5%)^28^ (purity: 93%, density: 880 kg/m^3^, molecular weight: 348 g/mol, Cytec Industries Inc., Canada); chitosan (medium molecular weight, acetylation degree: 0.23, batch number: BCCD5444, Sigma-Aldrich); glacial acetic acid (CH_3_COOH, 100%, Sigma-Aldrich); yttrium oxide (Y_2_O_3_, 99%, Alfa Aesar); gadolinium oxide (Gd_2_O_3_, 99%, Alfa Aesar); lanthanum oxide (La_2_O_3_, 99%, Alfa Aesar); nitric acid (HNO_3_, 65%, Alfa Aesar); and ammonium nitrate (NH_4_NO_3_, 98%, Fluka, Switzerland) were used.

### 2.2 PE Preparation

PE was obtained using a high-intensity ultrasound device (Hielscher, 200 W, UP200S, Germany). CS and Cy923 mixtures were placed in a double-walled glass vessel. The blends Cy923-CS were sonicated (90% amplitude) for 8 min at 10 °C by using a recirculating bath to maintain the temperature (Lauda, ECO RE 630 S, Germany).

CS solutions used in the experiments were obtained from a stock solution prepared by dissolving 7.5 g of CS into 500 mL (i.e., 15 mg/mL) of 1% (v/v) acetic acid at 40 °C and agitation at 1000 rpm. The resulting solution was filtered with a pore 3 glass filter and used within 1 week.

### 2.3 Adsorption Experiments

The adsorption tests were conducted in a batch regime. In general, a given amount (g) of freshly prepared PE was added to a volume (*V*) of an aqueous solution containing the REE to be recovered. A slight agitation was applied to ensure the complete homogenization, and the reaction was kept for 1 h at room temperature (22 ± 2 °C) without agitation. The PE was separated from the aqueous phase using centrifugal filters Amicon Ultra-15 by centrifugation at 2400*g* for 10 min. The specific information about the experimental conditions such as dose of PE (g/L), initial concentration (*C*_0_), *V*, and pH of the aqueous phase is provided in each section as well as in the caption for each figure.

The REE concentration was determined by optical emission spectrometry with inductively coupled plasma (ICP-OES, Optima 4300 DV, PerkinElmer). The removal (%) of Y was evaluated as the target value for the optimization experiments, which was calculated by eq 1, where *C*_0_ and *C*_*e*_ are the initial and final or equilibrium concentrations (mg/L), respectively.
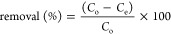
1

### 2.4 Optimization of PE Production

#### 2.4.1 Experimental Design

The amount of Cy923, the concentration of CS, and the pH of CS were the three factors evaluated by using the DoE approaches. Two statistical designs were applied sequentially, which included the following:

(i) Preliminary experiments (P-exps) consisted of a 2^3^ full factorial design. The target of these experiments was to determine the main effects and their interaction of the three factors evaluated in the PE formation. The factors and levels used in P-exps are shown in Table 1, and the detailed conditions used are presented in the Supporting Information (Table S1).
(ii) Optimization experiments (O-exps) applied a 3^3^ Box–Behnken design. The target of these experiments was to set the optimal conditions and the empirical mathematical model of the system according to eq 2. The information provided by P-exps was used to update the levels in O-exps. The levels used in O-exps are shown in Table 1, and detailed conditions used are presented in the Supporting Information (Table S2)

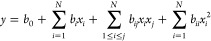
2where

**Table 1:** Factors and Levels Used in the P-Exps and O-Exps

			P-exps levels		O-exps levels		
label	factor	units	–1	–1	–1	0	+1
Cy923	Cy923 content	g	10.0	15.0	10.0	15.0	20.0
CS	CS concentration	mg/mL	2.0	10.0	5.0	10.0	15.0
pH	pH of CS	-	5.0	6.5	3.5	5.0	6.5

*y* = target value: yttrium yield

*x*_*i*_: factors: Cy923 content, CS concentration, and pH of CS

*N*: number of factors (3)

*b*_0_: ordinate section

*b*_*i*_, *b*_*ij*_, and *b*_*ii*_: regression parameters of linear, squared, and cross effects

The data analysis was performed using Design Expert software, version 11.1.2.0.

#### 2.4.2 Experimental Procedure

The extraction experiments were conducted according to Section 2.3. The specific experimental conditions included the following: dose: 10 g/L (dry basis), *C*_o_: 100 mg/L, *V*: 25 mL, and pH 3. The experiments were run by duplicate. Finally, with the resulted optimized material, the Y recovery was validated. The resulting material is named optimized pickering emulsion (oPE).

### 2.5 Characterization of the Optimized Pickering Emulsion (oPE)

The composition of oPE was established by quantification of water, Cy923, and CS (five replicates). The water content was measured in a Karl Fisher titrator (Mettler Toledo, V20, Germany) and by an infrared moisture analyzer (VWR, model: MB 6-M, Germany).

The content of Cy923 in oPE was determined based on the quantification of the phosphorus (P) present in Cy923 which initially is 0.0825 g. Before the P quantification, oPE was digested to extract and convert the organic phosphorus (P) into inorganic P, which allows the P determination by ICP-OES.^29^ In detail, ∼300 mg of oPE and 50 mg of NaNO_3_ were added to 25 mL of aqua regia (i.e., 6.25 mL of HNO_3_ 65% and 18.75 mL of HCl 37%) and digested in a microwave (CEM, Mars 5) at 5 °C/min and 190 °C for 20 min. The P present in the digestate was quantified by ICP-OES, and the Cy923 content was calculated and expressed in g Cy923/g oPE. The CS content was determined by the difference between the total mass and the determined masses of water and Cy923.

The stability of oPE was determined by analyzing the performance removing Y from an aqueous phase for 90 days, following the procedure described in Section 2.3, but at pH 2, which was the optimal pH from experiments planned in Section 2.6.1

Dynamic viscosity (η) measurements were carried out according to the methodology of Hohl et al.^30^ at shear rates from 0.1 to 1000 s^–1^ at 20 °C for 120 s. Dynamic moduli measurements related to the storage modulus (*G*′) and loss modulus (*G*″) were obtained by varying the angular frequency from 1 to 100 1/s at 2 Pa and 20 °C.

The surface functional groups were identified by Fourier transform infrared spectroscopy (FTIR spectroscopy, Spectrum Two IR, PerkinElmer). On the other hand, rheological measurements, including flow curves and dynamic moduli, were performed in a rheometer (Rheotest Medingen, RHEOTEST RN 4, Germany).

The morphology and size of oPE were analyzed by scanning electron microscopy (SEM, JSM 7001F, JEOL) after diluting and freeze-drying the samples. Optical microscopy (Keyence, VHX-5000) was used to visualize the oPE and droplet size. Volume distribution of oPE was measured by laser diffraction (LS) particle analyzer using the LS software (Beckman Coulter LS 13 320 v 6.01).

### 2.6 Application of oPE on REE Removal

#### 2.6.1 Influence of pH

The influence of pH in the Y removal performed by oPE was evaluated at pH ranging from 0.0 to 5.0. The lowest pH 0.0 was selected because it was visually observed that oPE started to be destroyed at pH < 1, while the maximum pH of experimentation was set at pH 5.0 to avoid Y precipitation. The experiments were set according to Section 2.3 (dose: 10 g/L (dry basis), *C*_o_: 100 mg/L, V: 25 mL). The pH of Y solutions was carefully adjusted using convenient HNO_3_ 0.1 or NaOH 0.1 mol/L. The Y removal yield was determined according to eq 1 and the equilibrium pH (pH_e_) was measured for further data analysis. The zero charge potential (pH_pzc_) of the material was evaluated, according to the methodology previously described.^31^

#### 2.6.2 Nitrate Media Effect

The influence of the nitrate concentration in the Y removal yield performed by oPE was evaluated. The nitrate concentration was varied from 0.06 mol/L (calculated from the HNO_3_ used in the solution preparation and corresponding to zero NO_3_^–^ added) to 2 mol/L. Ammonium nitrate was used to adjust the nitrate concentration. The experiments were conducted according to Section 2.3 (dose: 10 g/L (dry basis), *C*_o_: 100 mg/L, *V*: 250 mL, pH: 2). *C*_o_ was increased in comparison with the former experiments because an increase in the removal performance was expected.

#### 2.6.3 Kinetic Evaluation

The kinetic behavior of the metal removal reaction performed by oPE was studied. The experiments were conducted according to Section 2.3 (dose: 10 g/L (dry basis), *C*_o_: 1000 mg/L, *V*: 25 mL, pH: 2, [NO_3_^–^]: 1 mol/L). The kinetic data were adjusted using nonlinear equations of pseudo-first-order reaction (PFOR, eq 3), pseudo-second-order reaction (PSOR, eq 4), and Elovich model (eq 5).^32^
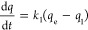
3
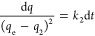
4
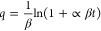
5where *q*_e_ (mg/g) is the equilibrium sorption capacity, *q*_1_ and *q*_2_ are the sorption capacity constants (mg/g) calculated by PFOR and PSOR, respectively, *t* (min) represents the time, *k*_1_ (1/min) and *k*_2_ (g/mg·min) are the PFOR and PSOR rate constants, respectively, α (mg/g·min) is the initial sorption rate, and β (dimensionless) is the desorption constant related to the extent of surface coverage and activation energy calculated in the Elovich equation.

#### 2.6.4 Equilibrium Isotherm Evaluation

The equilibrium isotherm evaluation consisted of varying the initial concentration of Y solutions from 0 to 1500 mg/L. The experiments were set according to Section 2.3. The *q*_e_ was calculated according to eq 6.
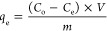
6where *V* is the volume (L) and *m* is the mass of the sorbent material in grams.

The equilibrium isotherms were modeled by the evaluation of nonlinear models of Langmuir, Freundlich, and Sips, according to eqs 7–9, respectively^33^
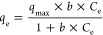
7
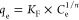
8
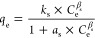
9where *q*_max_ (mg/g) is the Langmuir maximum sorption capacity, *b* (L/mg) is the Langmuir constant, *K*_F_ (mg/g)·(dm^3^/g*^n^*) is the Freundlich constant, and *n* (dimensionless) is the sorption intensity, and *k*_s_ (L/g), β_s_ (dimensionless), and *a*_s_ (L/mg) are the Sips constants of equilibrium, isotherm model exponent, and isotherm model constant, respectively. Additionally, the dimensionless constant *R*_L_ indicative of the separation factor or reaction favoritism was calculated by using eq 10.
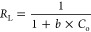
10

#### 2.6.5 Reusability Tests

To assess the Y recovery from the biomaterial, different adsorption–desorption cycles were conducted by using ethylenediamine tetraacetic acid (EDTA, 0.01 mol/L) as an eluting solution. The experiments were set according to Section 2.3. The desorption recovery or elution yield efficiency was calculated in eq 11, where *m*_A_ and *m*_D_ are the sorbed and desorbed masses of Y (mg), respectively.
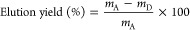
11

#### 2.6.6 Comparison with Liquid–Liquid Extraction

The consumption of Cy923 performed by L–L and oPE systems was evaluated to determine which system consumes less Cy923 during Y removal and in turn for determining the sustainable advantages of using PE in terms of green metrics. In both systems, Y solutions of 1 g/L, pH 2, and [NO_3_^–^] = 1 mol/L were used. The Y removal efficiency was calculated according to eq 2. The Cy923 concentration was varied from 0 to 1 mol/L.

L–L extraction experiments were conducted in separation funnels, in which 25 mL of the aqueous phase (containing Y) and 25 mL of the organic phase were added and agitated at 180 rpm for 20 min at room temperature. The organic phase consisted of Cy923 diluted in kerosene at the targeted Cy923 concentration. The funnels remained without agitation for phase separation, and the Y equilibrium concentration from the aqueous phase was measured. Distribution ratios (*D*) were calculated according to eq12.
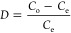
12

The experiments using oPE used to compare with the L–L system consisted of the addition of different quantities of oPE corresponding to the different concentrations of Cy923 into solutions of Y. The mass of PE added was calculated based on the content of Cy923 in oPE. The Y removal experiments were set according to Section 2.3. Distribution coefficients (*k*_d_) expressed in mL/g were determined by eq 13, where *V* (mL) is the experiment volume.
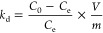
13

The green chemistry metrics evaluated were effective mass yield (EMY) and solvent intensity (SI) according to eqs 14 and 15 respectively.^34^ For EMY calculations, both kerosene and Cy923 were accounted.
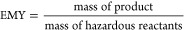
14
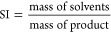
15

#### 2.6.7 Selectivity Studies

The capacity of oPE to selectively extract different REE from its main impurities was evaluated in three separated systems. The three separated blends included the following

(1) Y/Ca, as obtained from waste fluorescent lamp phosphor leaching,^3^
(2) Gd/Mg/Ca as obtained from (i) fluorescent lamp phosphor leaching^27^ and (ii) pharmaceutical residues,^1^ and
(3) La/Ni from nickel metal hydride batteries.^35^

Of these REEs, Y was considered representative for heavy REE, Gd was considered representative for medium REE, and La was considered representative for light REE. The initial concentrations were selected based on previous studies.^3,27,35^*k*_d_ from all elements was determined by eq 13.

#### 2.6.8 Application in a Real Scenario (Powder Lamp Waste)

Powder lamp waste (provided by Recyberica Ambiental S.L., Madrid, Spain) was used to evaluate the capability of PE to extract REE in a real scenario. To transfer the metals to an aqueous solution, a process previously developed by our research team consisting of solid-state chlorination, followed by soft leaching was applied. This process provides no strong pH conditions for the final aqueous liquor containing the metals. The methodology was adapted from Pavon et al.^3^ A mixture of around 1 g of fluorescent lamp powder and 1.5 g of NH_4_Cl were filled in a reaction flask set to a solid-state chlorination reaction (horizontal rotary kiln with a tubular inert gas connection) and heated to 285 °C for 3 h. The reactor was purged with nitrogen gas to remove toxic gases, and the system was cooling down at room temperature. The solid particles were transferred to a reaction flask and added to 25 mL of HNO_3_ of pH 2, and the liquid liquor was separated by filtration and used to the sorption experimentation using oPE under the experimentation conditions described in Section 2.3.

## 3 Results and Discussion

### 3.1 Optimization of PE Production

To establish the suitable conditions to achieve maximum REE removal efficiencies with the lowest reagent usage using PE, the optimal conditions for obtaining PE were studied. Two sequential experimental designs were applied according to Section 2.4. P-exps aimed to determine the main effects and interactions, while O-exps were applied to determine the optimal conditions for PE formulation based on the information provided by P-exps.

**Figure 1:**
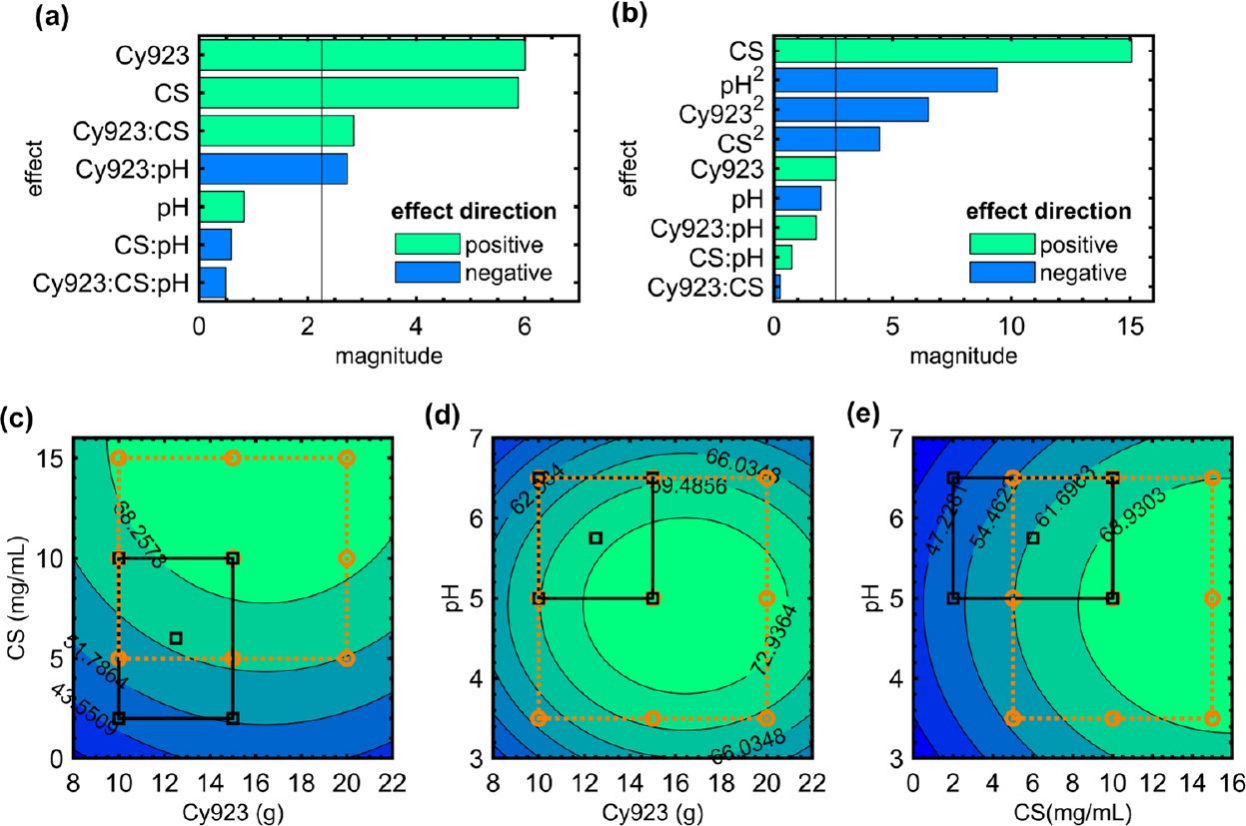
PE optimization. (a) Pareto plot of P-exps, (b) Pareto plot of O-exps, (c) contour plot of CS vs Cy923, (d) contour plot of pH vs Cy923, and (e) contour plot pH vs CS. Squares in continuous lines represent P-exps and dashed lines represent O-exps. (Experimental conditions: *C*_o_: 100 mg/L, pH: 3, a dose of PE: 10 g/L (dry basis), no agitation, *T*: room temperature.).

In P-exps, four out of seven factors evaluated presented significant effects on Y extraction yield (*p*-values <0.05 from analysis of variance (ANOVA) Table S3, Supporting Information) as shown in the Pareto diagram (Figure 1a). These four effects included Cy923 and CS as the main effects and Cy923:CS and Cy923:pH interactions. The Y recovery was positively influenced at higher concentrations of Cy923 and CS, while the pH had a minor effect at the evaluated levels. Besides, the interactions in which Cy923 was involved were statistically significant.

The results obtained from P-exps confirm the hypothesis that higher amounts of Cy923 extract a higher quantity of Y. Cy923 has been successfully tested for REE extraction;^21,23^ consequently, it plays the role of a functional molecule for recovering Y. In addition, higher CS concentration provides more interfacial area to accommodate more Cy923 into CS pickering particles. The pH of CS showed minor influence on Y uptake under the evaluated conditions in P-exports despite that the pH of CS was considered to significantly affect the CS properties (viscosity, state of chemical dissociation) and consequently its encapsulation performance.^36^ The role of pH in PE formation is better elucidated in the following sections.

Considering the information provided by P-exps, the levels of the factors evaluated were upgraded, and a new experimentation set was run using a 3^3^ Box–Behnken design (O-exps). This DoE was selected because this approach takes advantage of the central zone of a known optimal experimentation area reducing experimental efforts.^37^ Thus, Cy923 and CS concentrations were increased, and pH was decreased. The new O-exps were conducted according to Tables 1 and S2 (details of parameters used in O-exps, Supporting Information). The reduction of the pH (−1) level is the consequence of the negative influence of the interaction Cy923/pH shown in the Pareto diagram (Figure 1a) as well as to explore a possible quadratic effect or curvature in the optimal level not evidenced in P-exps.

The application of the 3^3^ Box–Behnken DoE resulted (Figure 1b and ANOVA Table S4, Supporting Information) in a quadratic equation model (eq 16) where the no significant effects (*p* > 0.05) were removed from the model. Therefore, eq 16 includes the main effects (Cy923, CS, and pH) and the quadratic effects (Cy923^2^, CS^2^, and pH^2^).
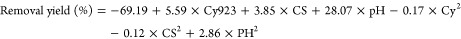
16

The equation model (eq 16) renders the maximum Y removal at 72.36%, using the following optimized parameters: 12.78 g of Cy923, 15 mg/mL of CS, and 4.89 as encapsulation pH.

The model was validated using oPE by 3-fold repeated experiments, which resulted in 72.59 ± 3.02% Y removal. Therefore, the quadratic empirical model in eq 16 represents the PE formation considering the evaluated factors.

In general, the levels used in P-exps and O-exps are depicted in Figure 1c–e, which show the contour plots and delimited areas of experimentation of both experimental designs applied. The performance of P-exps is presented in the region covered by the squares in solid lines, and the O-exps are delimited by dashed lines. Overall, a notable increment (∼29%) in Y removal was observed when Cy923 content and the CS concentration were increased (Figure 1c–e) reaching 73% of Y. Since the maximum Y removal was obtained, it is reasonable that no more Cy923 was encapsulated using 15 mg/mL CS. Unreacted Cy923 is visually observed in those O-exps in which the amount of Cy923 was the (+1) level of 20 g (Figure S1, Supporting Information). Note that the CS maximum concentration of 15 mg/mL was chosen as the (+1) factor level because increasing the pH of CS solutions over 5 was not possible at CS > 15 mg/mL due to its high viscosity (>270 mPa·s). On the other hand, the optimal area of pH is observable, when the (−1) level was decreased to 3.5 in O-exps. In addition, the factor interactions represented by the nonlinear contours in Figure 1d,e (Cy923:pH and CS:pH) confirm the role of pH in the PE formation. These results are in line with the literature reporting emulsions stabilized by CS, which can be controlled by tuning the processing conditions, such as pH and CS particle concentration.^36,38^

### 3.2 Characterization of oPE

#### 3.2.1 Composition, Stability, and Rheology

The composition by weight of oPE was water (64.3 ± 4.10%), Cy923 (32.2 ± 2.39%), and CS (4.99 ± 1.98%). Based on oPE composition, it was calculated that each gram of CS could accommodate 6.45 times its weight of Cy923. The 32.2% of Cy923 encapsulated represents an advantage compared to the 26% of Cy923 encapsulated in polysulfone by Ozcan et al.,^39^ obtained using a typical mixing method.

**Figure 2:**
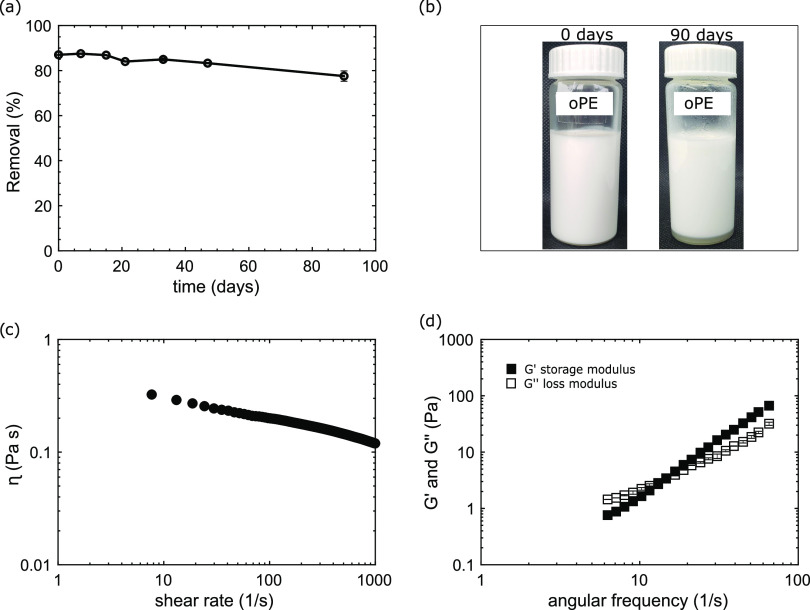
Composition, stability, and rheological properties of oPE. (a) Y removal yield stability over time (experimental conditions: *C*_o_: 100 mg/L, dose: 10 g/L (dry basis), *T*: room, pH: 2), (b) oPE appearance fresh and after 90 days of storage, (c) dynamic viscosity, and (d) dynamic modulus.

The stability of the oPE was assessed by the evaluation of Y removal and phase separation for 90 days. Y removal showed a slight decrease (∼7%) but remains above 80% (Figure 2a). Furthermore, the appearance of PE resulting from O-exps fresh prepared and after 90 days of storage at room temperature can be seen in Figure S2 (Supporting Information). In terms of phase separation, it is noted that a low CS concentration (5 mg/mL) results in higher phase separations, and PE with CS content from 10 to 15 mg/mL presented better stability. pH and Cy923 played a minor role in the stability observed after 90 days of storage.

Rheological properties allow determining the flow changes induced by aging, shear, and temperature and predicting the physical stability of oPE.^40^ The flow curve depicted in Figure 2c showed a decrease in the η value as long as the shear rate is increased. This shear-thinning behavior of oPE is indicative of its pseudoplastic behavior. Moreover, the storage modulus (*G*′) was larger than the loss modulus (*G*″) at a higher frequency range indicating that oPE is predominantly elastic (Figure 2d). The elasticity reduces the coalescence and consequently induces the long-term stability of oPE.^40^

#### 3.2.2 Surface Functional Groups and Interactions with REE

**Figure 3:**
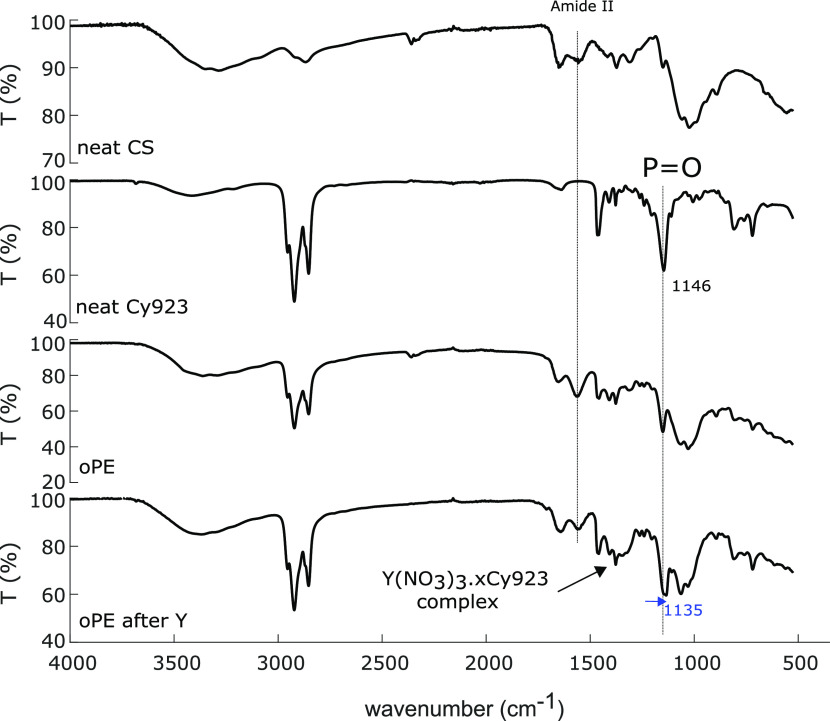
IR spectra of PE before and after Y removal and neat compounds CS and Cy923.

FTIR-ATR vibrational spectroscopy was used to identify the surface functional groups of the oPE and the interactions with Y after the removal as well as neat CS and Cy923 (Figure 3). In neat CS, we identified the groups related with O–H and N–H of primary amines at ∼3450 cm^–1^,^41^ and amide groups related with the presence of C=O, N–H, and C–N groups at stretching vibrations of amide I (1652 cm^–1^), amide II (1558 cm^–1^), and amide III (1317 cm^–1^), respectively.^42^ In neat Cy923, the sharp doublet stretching vibrations related to the C–H of alkyl chains associated with P=O at 2957 and 2865 cm^–1^ and the stretching vibration of P=O at 1146 cm^–1^ were determined.^43^ The oPE material showed a mix of the aforementioned stretching vibrations related to CS and Cy923. However, a relevant increase in the intensity of a peak related to the amide II of CS was observed in oPE (1558 cm^–1^), which is attributed to the interactions between the N–H groups of CS and the phosphine groups of Cy923. This observation is in line with previous works that observed this phenomenon in micro- or nanosized CS.^44,45^

After the contact of oPE with Y solution, a hypsochromic shift (from 1146 to 1135 cm^–1^) of the stretching vibration of P=O was observed as a consequence of the interaction P=O–Y. The participation of the P=O group was expected due to the high affinity of the oxygen atom of P=O of Cy923 to REE.^46^ In addition, the broadening in the zone between 1456 and 1315 cm^–1^ is related to the presence of a coordinate nitrate ion with Cy923 and Y to form the expected neutral complex Y(NO_3_)_3_·*x*Cy923 derived by the neutral mechanism exerted by the phosphine oxide donor group from Cy923, which act as strong Lewis base.^47,48^ Additional information about the complexation mechanisms is listed in Section 3.3.6. Furthermore, the peak at 1558 cm^–1^ (amide II) decreases in intensity after Y contact, which is a consequence of the CS participation in the Y removal.

#### 3.2.3 Size and Morphology

**Figure 4:**
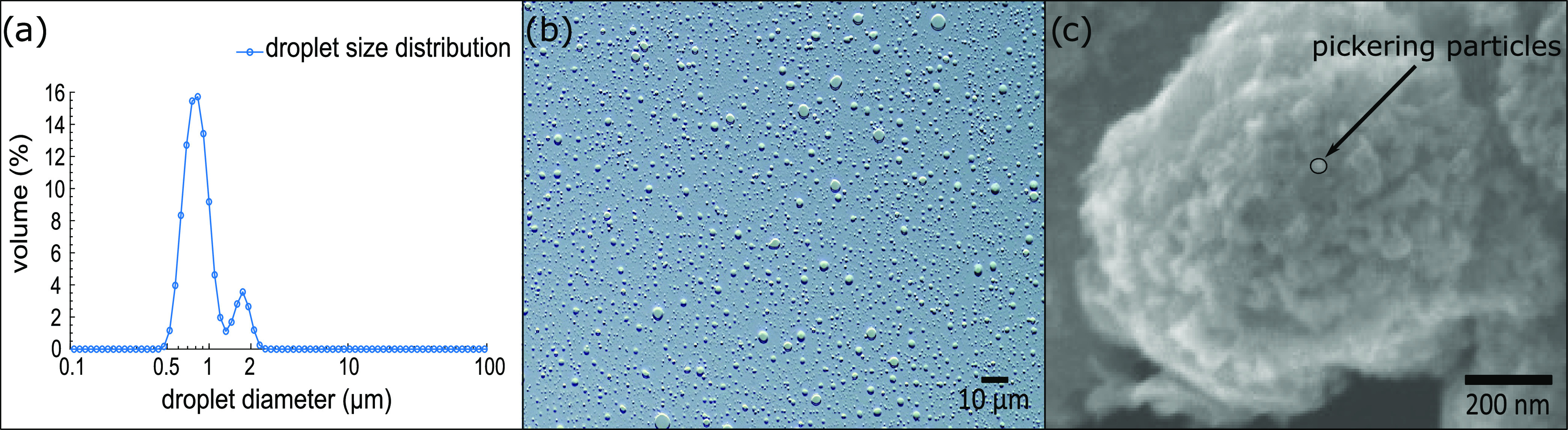
Droplet size (DS) distribution and microscope visualizations of oPE. (a) DS distribution, (b) optical microscopic view, and (c) SEM observation of the surface morphology of oPE.

oPE size analysis provided by the LS technique presented bimodal distribution, with a droplet size (DS) *D*_50_ of 0.83 μm and an average of 0.93 ± 0.34 μm (Figure 4a). Alternatively, optical microscopy observations (Figure 4b) showed the spherical shape of oPE and similar *D*_50_ of 1.10 ± 0.84 μm (*n* = 50 counts). The morphology of the oPE surface reveals that pickering particles of 40 ± 12 nm (*n* = 50 counts) cover the surface of oPE (Figure 4c). CS particles are absorbed in Cy923, forming a network of Cy923-CS nanoparticles, which in turn are assembled to form spherical microsized PE, similarly to previous studies dealing with PE.^49^

### 3.3 Application of oPE on REE Removal

The potential applicability of a sorbent material is given by its performance under conditions of pH, chemical species media, ion strength, and its capacity for recovering selectively target elements. In addition, aspects such as the determination of equilibrium kinetics, isotherms, and reusability are needed for conceptual process designs as well as to provide insight information on the sorption phenomena.

#### 3.3.1 Influence of pH

The pH of the aqueous phase, in which the sorption is conducted, determines the performance during metal removal. In addition, the physical resistance of materials can be compromised at different environmental stresses produced by different pH values.^50^ Although is well established that pH does not affect the performance of solvating EM such as Cy923,^51^ it is also well established that pH strongly affects the sorption performance of CS.^52^ Thus, an evaluation of the influence of pH on the performance of oPE for Y removal was conducted using harsh acidic conditions to simulate a possible real aqueous environment.

**Figure 5:**
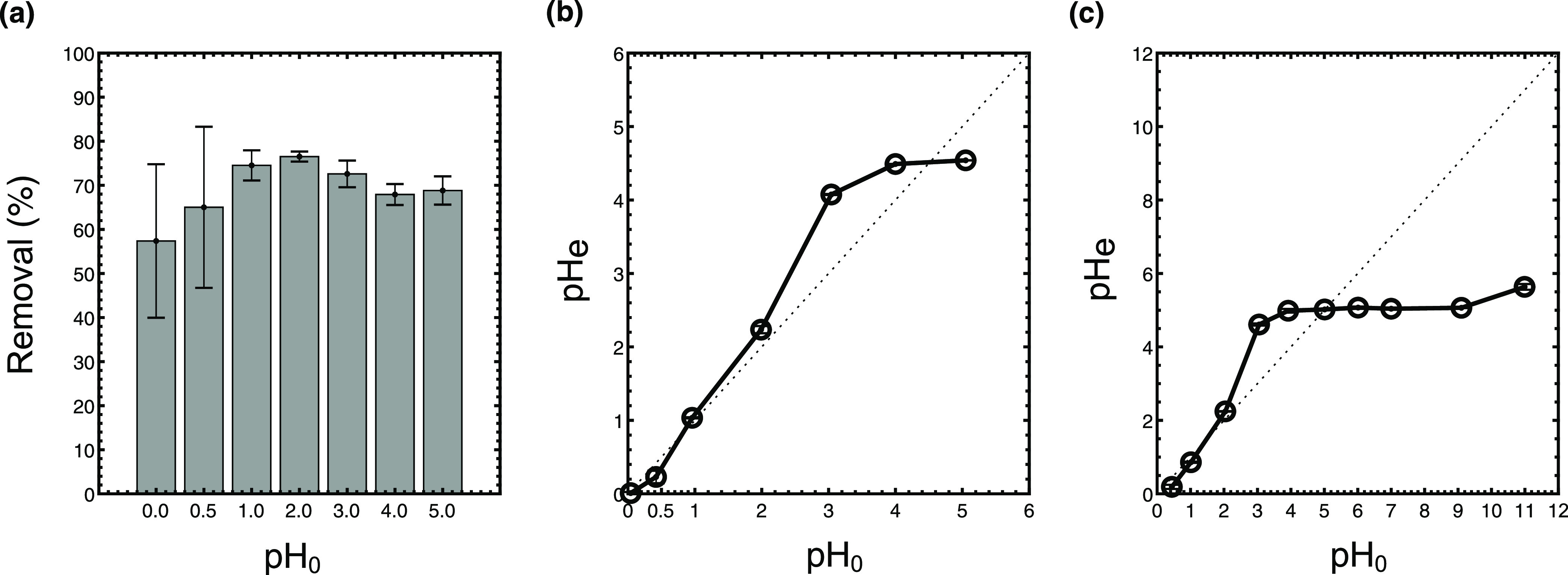
Influence of the pH in Y removal using oPE. (a) Y removal at different pH_0_, (b) changes in pH after sorption (experiment conditions: *C*_o_: 100 mg/L, dose: 10 g/L (dry basis), *T*: room, no agitation), and (c) zero charge evaluation of oPE.

The best Y removal performance was reached at pH 2.0, obtaining Y recoveries up to 76.5% (Figure 5a), while the performance dropped at pH < 1, and a slight decrease was revealed at pH > 3.

The lower Y removal performance at pH < 1 was caused by the harsh acidic conditions to which the oPE were exposed. At those pH values, oPE was destroyed, which was observed by the naked eye (Figure S3, Supporting Information). In addition, the droplet sizes and droplet size distributions of the oPE after each experiment at different pHs were measured (Table S5 and Figure S4), observing at pH 0 and pH 0.5 bigger droplet sizes (*D*_50_) (56 and 31 μm) and bigger dispersions than at pHs from 2 to 5, which present lower particle sizes (1.6 μm in average) and lower size distributions. The bigger droplet sizes and distributions at low pHs are expected due to the strong repulsive forces caused by protonation of amino groups of CS,^53^ which cause agglomeration and droplet breaking. Besides, the bigger droplet sizes are consistent with the lower results of the extraction performance at low pHs, while lower droplet sizes at higher pHs perform better removal of Y. In addition, a slight increase in the droplet size was observed compared with the original oPE from 0.8 to 1.0 μm at pH ≥ 2, which did not result in a decrease in the extraction performance.

In terms of removal performance, the oPE presented stability at pH 1 or 2; in spite of that, CS at this pH values normally causes that amine groups of chitosan to tend to protonate and become positively charged, which will pose a strong repulsion to cations due to electrostatic repulsion. However, oPE presents physical stability at pH 1 or 2 and excellent Y removal due to the nature of the emulsion formed. Several literature studies have reported the stability of pickering emulsions based on CS against lower pHs.^54^

The changes in the pH of the aqueous phase during the sorption process (Figure 5b) help to explain the change in the Y removal at pH ≥ 1. The pH_e_ remains without notable changes at pH < 2.0, followed by positive shifts at 2.0 ≤ pH ≤ 4.6 and negatives shifts at pH ≥ 4.6 (Figure 5b). A similar tendency is observed in the pH_pzc_ profile of the oPE (Figure 5c). pH_pzc_ is the point where the net surface charge of a material is zero,^55^ and the behavior of pH change along a range of pHs reflects the surface charge of the material. The pHs over and under the diagonal dashed line (Figure 5c) demonstrate a net charge on the surface material, pHs over the dashed line are related to the positive surfaces, and pHs under dashed lines represent negative net surface charge. oPE do not show a change in the pH < 2. It is reasonable that no changes in the pH_e_ were produced at pH < 2.0 because Cy923 is a neutral extractant whose mechanism is based on complexation between REE, extractant, and anion media,^51^ while the changes over pH 2 are related to the influence exerted by CS. The surface functional groups of CS can be protonated or deprotonated depending on the p*K*_a_ of the functional groups.^52^ Thus, oPE becomes positively charged at 2.2 ≤ pH < 5.1, and over its pH_pzc_ (5.1), the material becomes negatively charged. However, the effect of CS protonation does not compromise its removal performance because oPE is mostly composed of Cy923.

In general, oPE based on CS-EM can perform recoveries from pH ≥ 1 until the pH of metal precipitation. CS-based sorbent materials are physically destroyed or present low sorption capacities at pH < 2.0 due to the CS functional groups being mostly protonated.^31,56^ The oPE developed here showed resistance to the destabilization phenomena at acidic conditions of pH 2 and with certain risks, even at pH 1.

#### 3.3.2 Nitrate Media Effect

The nitrate concentration can play a role in the removal of Y as well as in the physical stability of PE for several reasons, among which

- Nitrate concentration influences the removal performance when metal-salt extractants such as Cy923 are applied.^57^

- The ion strength produced by the increased quantity of nitrate ions affects the physical stability of emulsions.^58^

**Figure 6:**
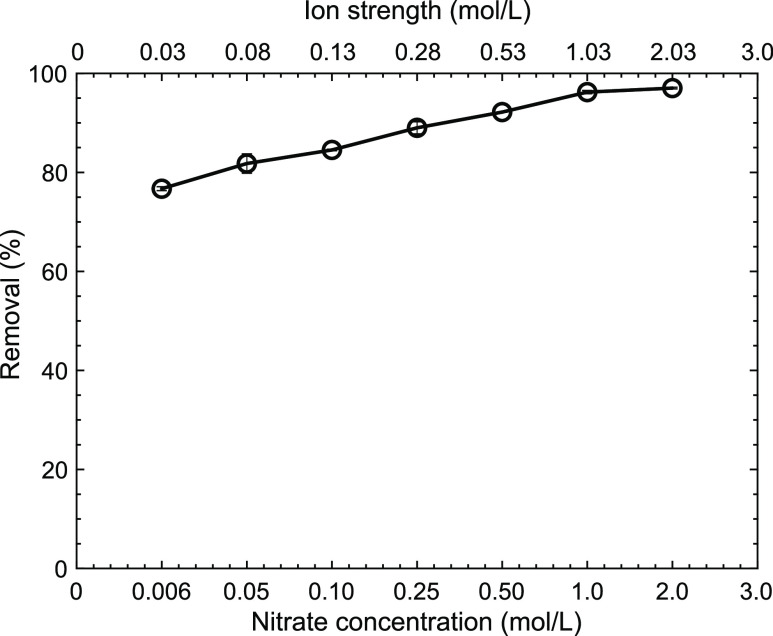
Effect of nitrate concentration on the Y removal performance. (Experimental conditions: *C*_o_: 0.25 g/L, pH_o_: 2.0, oPE: 10 g/L, *T*: room, no agitation.).

Figure 6 shows the influence of the nitrate concentration on the Y removal yield. The latter was observed to constantly increase as the nitrate ion concentrations were raised to 1.0 mol/L until the removal efficiency remains at its maximum value of ∼96% until 2 mol/L. The Y removal yield increase upon scaling the nitrate ion concentration is a behavior usually observed using solvating extractants because a fixed number of anions are needed to complete the complexation reactions.^57^ Thus, at higher nitrate ion concentration, larger extractability was observed.

On the other hand, oPE resists (Figure 6) high ion strength (>2 mol/L). Usually, PE is affected at high ion strengths,^59^ but in the case of the material evaluated hereby, the performance of the material for Y removal was not influenced, and additionally, no Cy923 leaking (oily) was observed until the ion strength of 2.03 mol/L is reached, which confirms the resistance of oPE to the stress applied.

Nitrate ions might be found in the aqueous phase rich in metals when nitric acid is used during the leaching process. Acid leaching is usually applied to transport the metals from the solid minerals or e-wastes to the aqueous phase before metal separation or removal. In addition, nitrate ions could be added during the solid–solid extraction process, such as solid-state chlorination. Alternatively, nitrate ions are required to improve the coordination ability of Cy923 even when other anions are present.^3,60^ The application of oPE is feasible both in nitric acid or solid-state because the oPE withstand the ion strength 0.2–2.0 mol/L of these industrial processes,^22^ in both scenarios.

#### 3.3.3 Kinetics Evaluation

**Figure 7:**
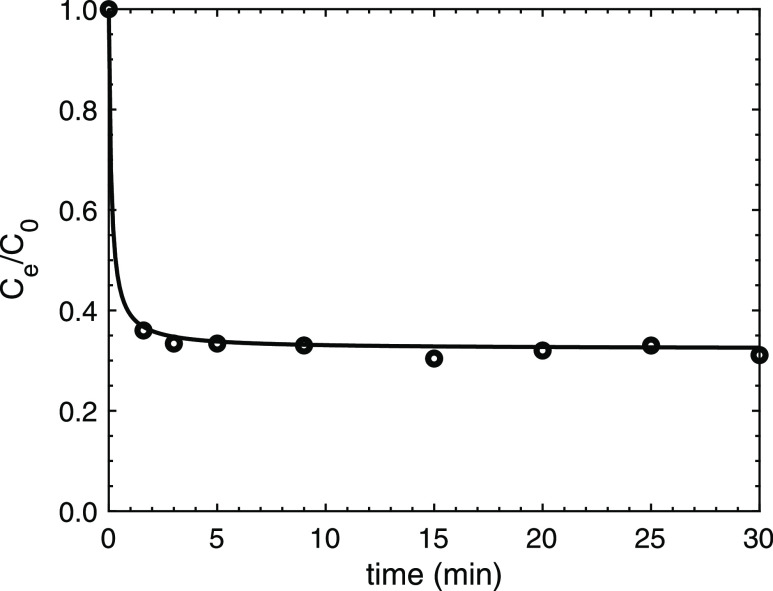
Kinetics of Y removal using oPE. (Experimental conditions: *C*_o_: 1000 mg/L, pH: 2.0, oPE dosage: 10 g/L, *T*: room, no agitation, [NO_3_^–^]: 1 mol/L) Solid line: modeled line by the PSOR model.

The kinetic studies provide key information for the conceptual designs of reactor units and give extra theoretical physicochemical knowledge of the process.^61^ oPE reaches the removal equilibrium in less than 5 min (Figure 7). The reaction times taken by the process indicate that PSOR fits better than PFOR and Elovich (Table S6 in the Supporting Information). Therefore, the metal uptake is of second order with respect to the surface sites available for the Y removal.^32^ The observed equilibrium time shows a remarkable advantage over extractant-polymer-based materials (Table 2) which was similar to those observed in L–L extraction. Sorbent materials for Y recovering required long equilibrium times (hours)^9,62^ that are significantly larger than the 5 min of oPE developed herein. However, the kinetics of our current oPE system is similar to L–L systems.^63^ In addition, oPE can be compared with materials developed to SPE^10,11,64^ and developed for REE selective enrichment, with presented excellent selectivity, good sorption capacity, and moderate kinetics.

**Table 2:** Comparison of oPE REE Removal Performance with Similar Materials

material	target element	*q*_max_* (mg/g)	equilibrium time (min)	reference
PE-based on Cy923 and CS	Y	90	<5	current work
D2EHPA encapsulated in PES, PVA, and carbon nanotubes	Y	44	240	(9)
PC88A encapsulated in PES and PVA	Y	100	322	(62)
poly(carboxymethyl)-cellulose	La	170	150	(64)
Ion imprinted La(III)IP-CS/PVP	La	39.34	120	(10)
straw-supported ion imprinted polymer (straw@IIP)	La	125	9	(11)
Amberelite XAD-4 impregnated with Aliquat-336	Gd	4.44	30	(65)
styrene divinylbenzene resin	Gd	15.49	60	(66)
	Tb	24.93		

D2EHPA: di(2-ethylhexyl) phosphoric acid, PES: polyether sulfone, PVA: poly(vinyl alcohol), divinylbenzene resin, PC88A: 2-ethylhexyl ethylhexyl phosphonic acid, PVP: poly(vinyl pyrrolidone). *Maximum sorption capacity calculated by the Langmuir model.

The emulsion nature and reduced droplet size (∼1 μm) of oPE permit a quick and complete miscibility with an aqueous phase and provide an extended surface contact between the oPE material and the metal to be recovered. The fast kinetics performed by oPE represents shorter retention times within reactors and consequently reduced reactor volume, which means reduced costs at the industrial level. Furthermore, continuous flow microchannels or microreactors seem a feasible technique in which PE can be implemented at the industrial scale.

#### 3.3.4 Equilibrium Isotherm Evaluation

**Figure 8:**
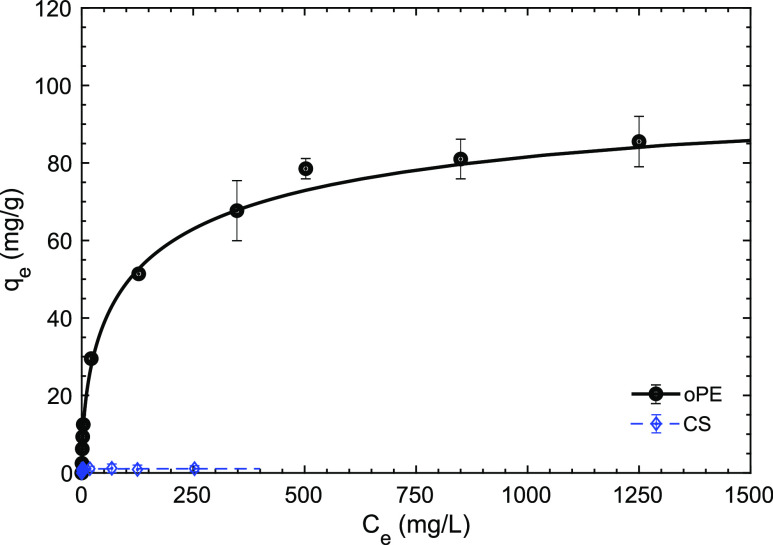
Equilibrium isotherm of Y and using oPE and CS. (Experimental conditions: pH_0_: 2.0, oPE and CS dosage: 10 g/L (dry basis), *T*: room, no agitation, [NO_3_^–^]: 1 mol/L.).

The maximum sorption capacity of sorbent materials and the phenomenon governing the liquid–solid separation are evaluated by the equilibrium isotherms and their related mathematical modeling.^67^ This evaluation aimed to determine the maximum sorption capacity of oPE to recover Y (Figure 8). The plateau representing the material saturation or the maximum sorption capacity was presented at *q*_e_ ∼ 80 mg/g. A steep slope is observed at the initial part of the isotherm, which is indicative of the higher affinity of oPE for Y.^68^ Furthermore, neat CS was also evaluated, reaching *q*_e_ of ∼1 mg of Y/g, which confirms the negligible participation of CS in Y removal at the experimental conditions used (Figure 8). The low *q*_e_ of CS is explained by the high protonation of CS surface functional groups and, in turn, a weak physical resistance of CS at working pH conditions.

The SIPS model fits better the experimental data than Langmuir and Freundlich models (Table S7, Supporting Information). The SIPS model, which is a combination of Langmuir and Freundlich models, establishes that heterogeneous adsorption is governing the REE extraction.^33^

This sorption is produced because the molecules of Cy923 are incorporated into the tiny particles of CS, creating a heterogeneous structure from which the main participation in the sorption is conducted by Cy923. According to the results obtained from eq 10, which indicates the favoritism of the sorption when (0 < *R*_L_ < 1),^69^ the factor *R*_L_ of 0.07, calculated at *C*_o_ of 1 g/L, indicates that oPE performs favorable sorption. Likewise, oPE had comparable or higher *q*_max_ compared to similar sorbent materials (Table 2), including capsules based on polymers impregnated with extractant molecules such as D2EHPA, PC88A, Aliquat-336 used to extract Y^9,62^ and Gd,^65^ resins,^66^ or materials used in SPE for La concentration.^10,11,64^ Thus, oPE is suitable to extract Y with competitive max values. The capabilities to extract another REE are shown in the next sections.

#### 3.3.5 Reusability Tests

**Figure 9:**
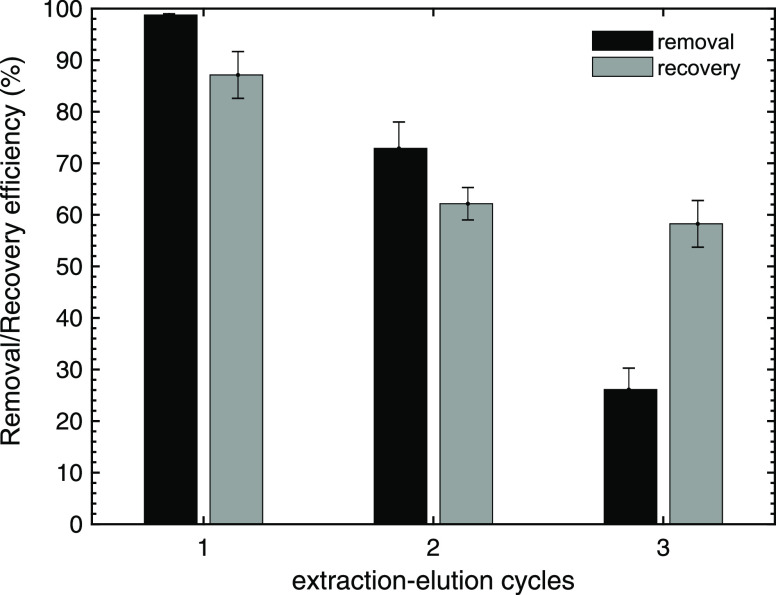
Evaluation of oPE reusability. (Experimental conditions: *C*_o_: 100 mg/L of Y, pH: 2.0, PE dosage: 10 g/L (dry basis), *T*: room, no agitation, [NO_3_^–^]: 1 mol/L, ion strength: 1 mol/L; Elution: EDTA 0.01 M of pH 5.5.).

The capacity of oPE for reuse after several adsorption–desorption cycles is presented (Figure 9). The elution after one usage reached 87% of Y recovery. However, after the first usage, a notorious decrease in the oPE capability for extracting Y was observed. The recovery yield fell from 99 to 26% along with the 3 utilizations. On the other hand, the elution performance achieved 87% in the first cycle; however, it fell to ∼60% in the next oPE usages.

The recovery decrease is correlated to the low stability of emulsions under the conditions of ultrafiltration and elution. Therefore, the use of the studied oPE should be limited to two cycles and recovering Cy923 for the preparation of a new oPE. PE can be the base for developing reinforced PE microcapsules to be applied in continuous flow column reactors. However, the strengthening of the PE shell must be properly addressed. Several techniques, including cross-linking combined with drying alternatives, are currently addressed by our research group as a strategy for reinforcing the mechanical resistance of PE.

#### 3.3.6 Comparison with Liquid–Liquid Extraction

**Figure 10:**
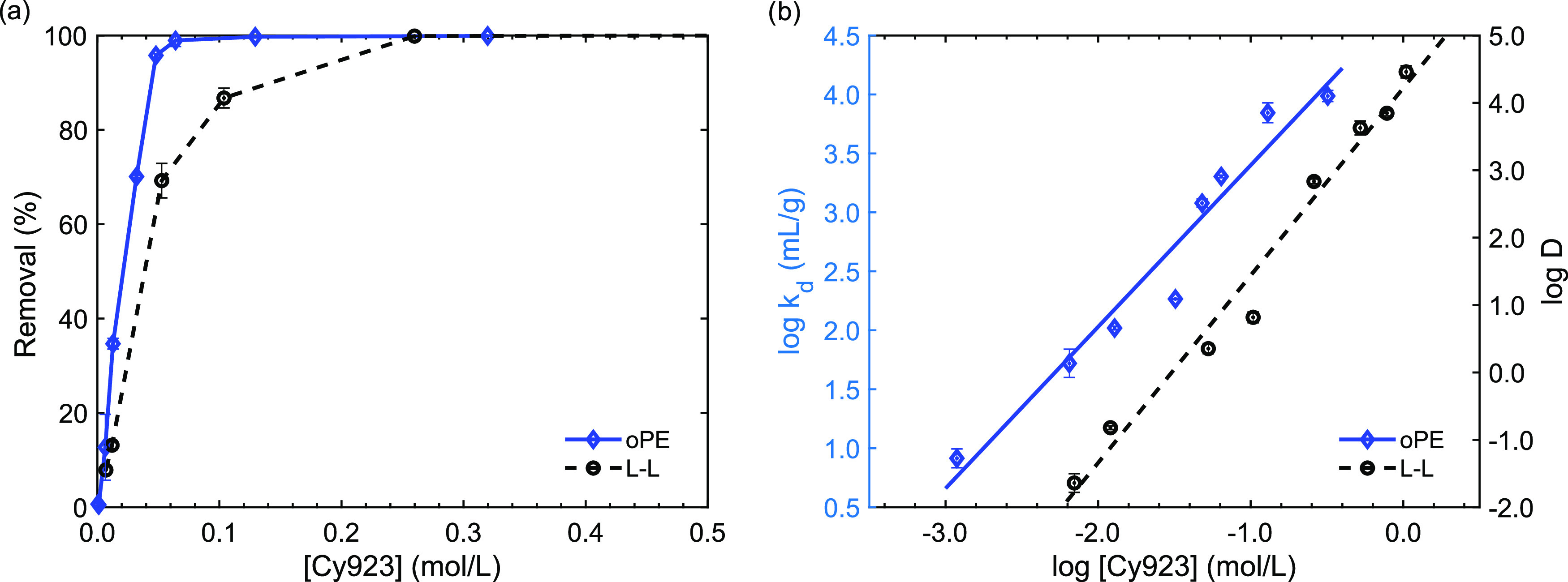
Comparison of L–L and oPE systems. (a) Y removal of oPE vs L–L. (b) Separation factors dependence of Cy923 concentration. (Experimental conditions: *C*_o_: 1 g/L, pH = 2.0, [NO_3_^–^] = 1 mol/L, *T*: room, no agitation for oPE system, and 180 rpm for L–L system.).

The herein developed oPE and the L–L method were compared under the same conditions to evaluate the consumption of Cy923 correlated with the removal yield. These results were also used to determine green metrics for assessing the sustainability using oPE. When oPE was applied, 0.14 mol of Cy923 was needed to extract 99.7% of Y, and while applying the L–L process, 0.27 mol of Cy923 was used to extract Y with 99.8% of extraction efficiency (Figure 10a). Thus, using oPE, 48% of Cy923 was saved compared with the consumption done by L–L extraction, which consequently reduces the costs associated with the use of oPE compared with L–L extraction.

In terms of green metrics, the sustainability of the oPE application against L–L extraction can be assessed by the EMY calculus (eq 14). The calculated EMY was 3.10 for oPE and 10.64 for L–L extraction. Thus, the application of oPE resulted in ∼3 times less hazardous substances usage than L–L extraction. For this particular experiment comparison, EMY was considered as the most suitable metric compared with another widespread green metrics such as *E*-factor or atom economy.

Additionally, the number of Cy923 molecules needed for the complex formation was determined using the slopes resulting from the linear analysis of the plots *k*_d_ and *D* vs Cy923 concentration (Figure 10b).^70^ This analysis determined that 1.21 (∼1) and 2.79 (∼3) mol of Cy923 were involved in the Y removal by oPE and L–L, respectively. Thus, the equilibrium expressions of oPE and L–L systems are defined in eq 17 and 18, respectively.
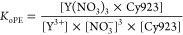
17
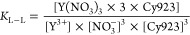
18

The less consumption of Cy923 by oPE was observed because the extended contact area provided by the small capsules coupled with the complete homogenization between the reagents allows an enhanced efficiency of Cy923 compared to L–L extraction. In addition to the less consumption of Cy923, the application of oPE provides extra advantages over the traditional L–L technique such as the following:

i. No need for solvents (e.g., kerosene, dodecane, etc.). L–L extraction required 0.9 m^3^ of kerosene by each m^3^ of Y solution of *C*_o_ of 1 g/L (i.e., 0.25 mol/L of Cy923 in organic phase), while using PE, organic solvents are prevented. In terms of green metric SI (calculated by eq 15), oPE is the greener alternative as SI is 0.0, while L–L results in SI of 0.18. Evidently, the no use of kerosene represents a sustainable advantage achieved using the oPE alternative.
ii. Reduction in costs using oPE compared with L–L extraction. To process 1 m^3^ of aqueous solution containing 1 g/L of Y (i.e., Figure 10 conditions), the cost associated with using oPE is ∼3000 USD while with L–L extraction is ∼5100 USD (details in Table S8). This is approximately 40% in cost reduction. However, further experimentation and full cost analysis in real conditions must be addressed for each particular situation.
iii. No need for agitation for reaction in opposition to L–L extraction. The emulsion-like nature of oPE permits a complete dispersion and homogenization of PE into the aqueous phase, which allows for saving energy and cost of the rotators.

#### 3.3.7 Selectivity Studies

A key aspect of the industrial application of sorbent materials is related to the capacity of recovering selectively a target metal from aqueous solutions. To evaluate the selectivity of oPE for REE removal, three systems consisting of blends of rare earths and their associated main impurities were tested. The evaluated systems correspond to three typical urban wastes including a lamp powder waste, a pharmaceutical residue, and an end-of-life Ni-MH battery and are particularly focused on the separation of the representative rare earth from the main non-REE found in these wastes. The blend compositions and initial concentrations corresponding to the aqueous phases are reported in the solid–liquid extraction literature in real samples.^27,35^ The initial concentration values and experimental results are presented in Table S9 (Supporting Information).

**Figure 11:**
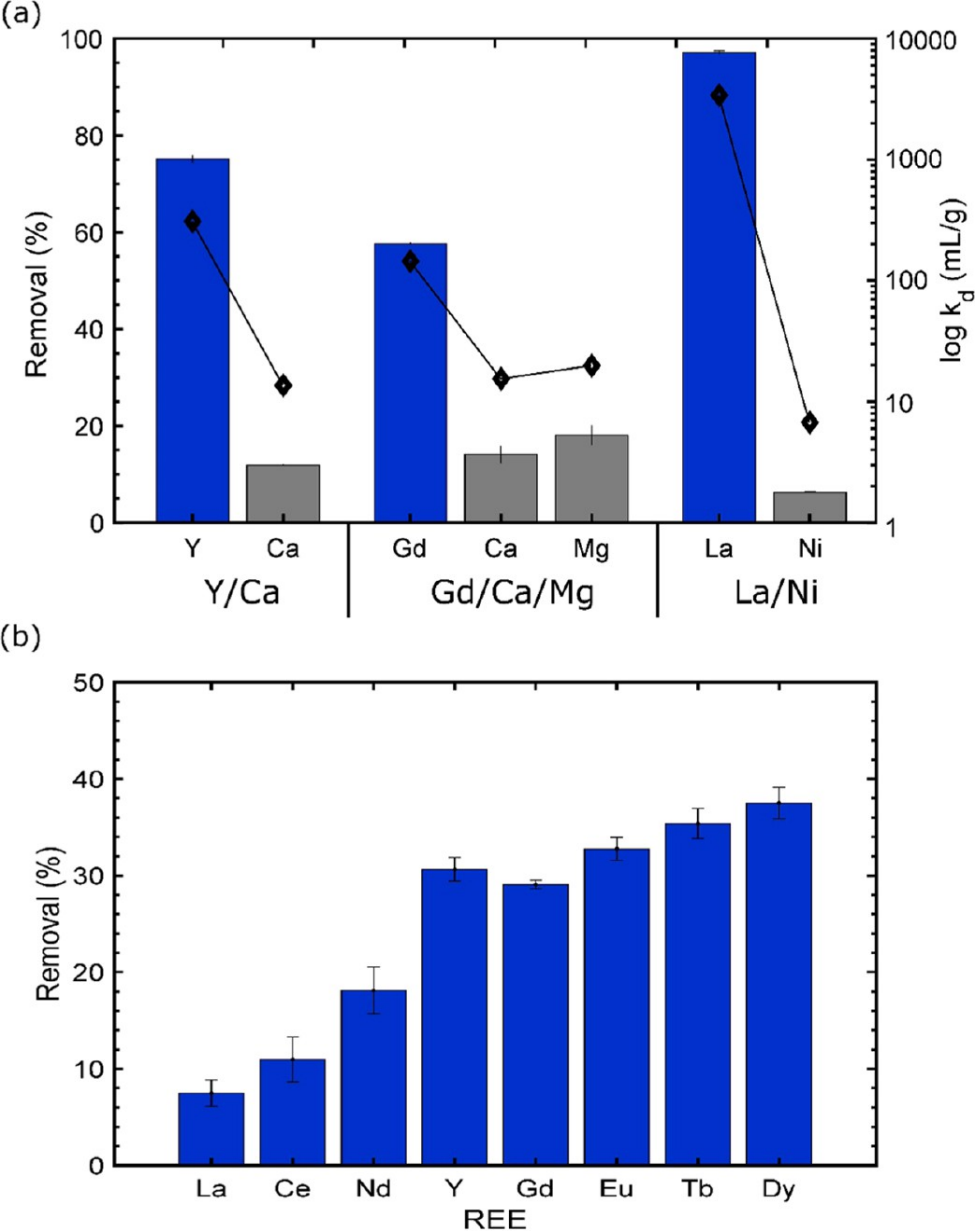
Selective removals. (a) Performance by oPE in three blends: Y/Ca, Gd/Mg/Ca, and La/Ni systems. Bars correspond to removal yields (left *y* axis) and diamond plots correspond to *k*_d_ (right *y* axis). (Experimental conditions: pH = 2.0, [NO_3_^–^] = 1 mol/L, oPE dose: 10 g/L (dry basis), *T*: room, no agitation). (b) performed by oPE in a mix solution of REE. (Experimental conditions: *C*_o_: 100 mg/L of each REE, pH = 2.0, oPE dose: 10 g/L (dry basis), *T*: room, no agitation.).

The significantly higher removal REE yield and *k*_d_ observed for REE compared with the non-REE (Figure 11a) confirm that oPE has the capacity for selectively extracting the REE from the main impurities found in urban wastes.

These results showed in Figure 11a were expected due to the Cy923 molecule that builds the oPE selected based on the high affinity observed for Y,^22^ Gd,^22^ or La^60^ using the L–L process. The efficient selectivity for REE performed by oPE confirms that the simple approach of encapsulating a probed EM into a sustainable polymer does not affect the selectivity performance of the selected Cy923 EM and represents an easy route to give functional capabilities to a sorbent material.

In addition, oPE was able to remove the REE evaluated among a mix of them, presenting an ascending tendency in the removal efficiency according to the molecular weight of the REE, with the exception of Y. Light REE (La, Ce, Nd) and Y presented removal efficiencies of less than 30%, while heavy REE (Gd, Eu, Tb, Dy) presented efficiencies of removal from 30 to 37% (Figure 11b). The differences in the range of removal efficiencies achieved in experiments shown in Figure 11b compared with Figure 11a are attributed to the quantity of elements in the mix solutions, which affect the sorption capacity of the oPE material. However, it has to be noted that chemical conditions used to the extraction experiments will define the separation among REE, particularly the chemical media and its concentration (e.g., Cl^–^, NO_3_^–^, etc.).^21^ Thus, to set the optimal conditions to separate the REE, several studies involving more factors and process options are mandatory. The evaluations presented in this section demonstrate the capability of oPE to separate REE from cations found in a synthetic solution with a composition from real problems related to REE recycling and for removing REE among a mixture of them.

#### 3.3.8 Application of oPE in a Real Scenario (Fluorescent Lamp Powder Waste)

**Figure 12:**
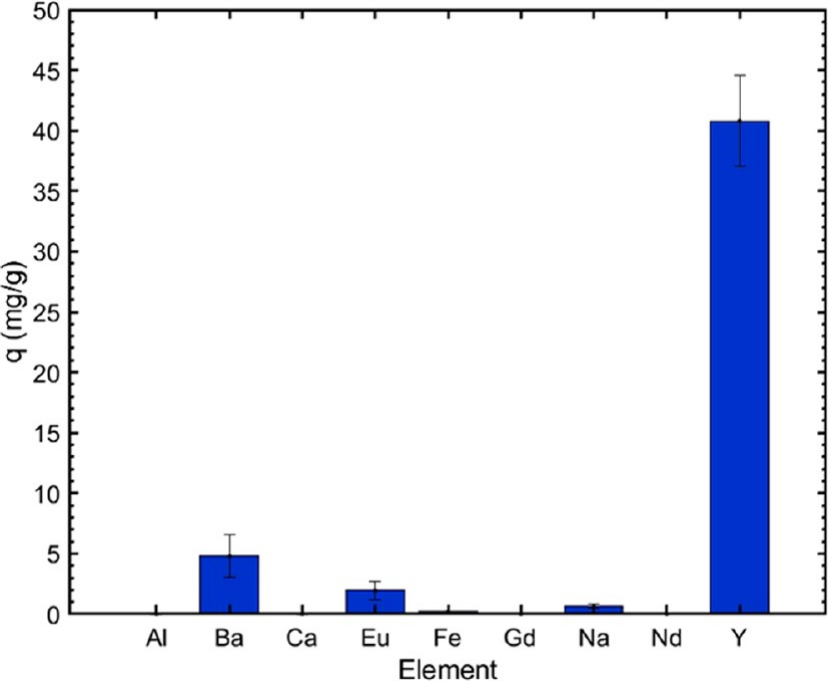
Performance of oPE in a real aqueous solution leached from a fluorescent lamp powder using the solid-state chlorination extraction process, followed by leaching with HNO_3_ 0.01 M. (Experimental conditions: *C*_o_: Table S10, pH = 2.0, oPE dose: 10 g/L (dry basis), *T*: room, no agitation.).

The capability of oPE to extract REE from a real case of fluorescent lamp powder waste was tested. The powder was processed by solid-state chlorination, followed by soft leaching using HNO_3_ of pH 2 (0.01 M). The resulting liquid liquor, before applying oPE, presented Y and Eu as mainly REE, combined with other elements, including Ba, Na, Ca, Al, Fe, Nd, and Gd (Table S10). The sorption step using oPE presented excellent selectivity to Y (Figure 12 and Table S10). The mass of Y was transferred to oPE preferably over the other elements presented in the liquor problem, thus demonstrating the capability of oPE to extract Y in a real scenario. The sorption capacity of oPE applied in this experiment presents a decreasing respect to the evaluation in a synthetic solution with pure Y (Figure 8), from 90 to 40 mg/g, which is expected to the participations of more ions in the extraction process. However, oPE maintains its selective performance toward Y ions. To improve results, several further experiments must be conducted to fix the optimal conditions to removing, eluting, and purifying the total of the valuable REE from the aqueous phase.

## 4 Conclusions

PE has been demonstrated to be a feasible material for selective removal of REE from the aqueous phase. The experimental design approaches used in this study establish the optimal conditions for PE synthesis. The microsized PEs were demonstrated to be stable for 90 days and to be able to perform high removal yields efficiencies, supporting low pH, high nitrate, and ion strength concentrations, presenting fast kinetics and acceptable sorption capacity. PE also showed advantages compared with the L–L process, including less consumption of Cy923, no use of organic diluents, and no agitation needed for the removal process. In addition, PE showed selectivity to REE in synthetic and real cases of extraction. PE is a sorbent material with simple and fast manufacture and is presented as a sustainable alternative for REE removal.

## Supporting Information Available

Experimental designs applied, statistical analysis from experimental designs, droplet size determination and droplet size distribution of PE at different pHs of the aqueous phase, parameters obtained from modeling of equilibrium kinetics, parameters obtained from modeling of equilibrium isotherms, cost analysis, details of the selectivity test, details from the application of oPE in a real case, XRF analysis of fluorescent lamp powder, visual observations of PE fresh and after 90 days of storage (PDF)
