# Supplementary material for: Bioderived Pickering Emulsion Based on Chitosan/Trialkyl Phosphine Oxides Applied to Selective Recovery of Rare Earth Elements
Source: ACS Appl Mater Interfaces. 2023 Dec 13;15(51):59731–45. doi: 10.1021/acsami.3c10233 (PMC10802976; doi:10.1021/acsami.3c10233)
Supplement: Supplementary file 1 — am3c10233_si_001.pdf [file am3c10233_si_001.pdf]

Supporting information

Bioderived pickering emulsion based on chitosan/trialkyl phosphine oxides applied to selective recovery of rare earth elements

Byron Lapo*[a,b,d], Sandra Pavón[c,d], Javier Hoyo[e], Agustín Fortuny[f], Martin Bertau[c,d], Paul Scapan[d] and Ana María Sastre[a]

a. *Department of Chemical Engineering, Universitat Politècnica de Catalunya, ETSEIB, Diagonal 647, 08028 Barcelona, Spain*
b. *School of Chemical Engineering, Technical University of Machala, UACQS, BIOeng, 070151 Machala, Ecuador.*
c. *Fraunhofer Institute for Ceramic Technologies and Systems IKTS; Fraunhofer Technology Center for High-Performance Materials THM, Am St.-Niclas-Schacht 13, 09599 Freiberg, Germany.*
d. *Institute of Chemical Technology, TU Bergakademie Freiberg, Freiberg, Leipziger Straße 29, 09599, Germany*
e. *Department of Physical-Chemistry, Universitat de Barcelona, 08028 Barcelona, Spain*
f. *Department of Chemical Engineering, Universitat Politècnica de Catalunya, EPSEVG, Av. Víctor Balaguer 01, 08800 Vilanova i la Geltrú, Spain*

TABLES

Table S1. Specific conditions used in P-experiments (P-exps)

Label	Standard number	Run number	Cyanex content (g)	Chitosan concentration (mg/mL)	pH
S1	1	8	10	2	5
S2	2	5	15	2	5
S3	3	3	10	10	5
S4	4	6	15	10	5
S5	5	1	10	2	6.5
S6	6	4	15	2	6.5
S7	7	2	10	10	6.5
S8	8	7	15	10	6.5

Table S2. Specific conditions used in O-experiments (O-exps)

Label	Standard number	Run number	Cyanex content (g)	Chitosan concentration (mg/mL)	pH
O1	1	4	10	5	5
O2	2	13	20	5	5
O3	3	8	10	15	5
O4	4	1	20	15	5
O5	5	6	10	10	3.5
O6	6	15	20	10	3.5
O7	7	5	10	10	6.5
O8	8	14	20	10	6.5
O9	9	7	15	5	3.5
O10	10	10	15	15	3.5
O11	11	2	15	5	6.5
O12	12	3	15	15	6.5
O13	13	9	15	10	5
O14	14	11	15	10	5
O15	15	12	15	10	5

Table S3. ANOVA for full factorial model (P-exps)

Source	Sum of squares	df	Mean square	F-value	P-value	
**Model**	174.07	5	34.81	57.62	0.0171	significant
A-Cyanex	72.26	1	72.26	119.60	0.0083	
B-Chitosan	69.17	1	69.17	114.48	0.0086	
C-pH	1.38	1	1.38	2.29	0.2697	
AB	16.29	1	16.29	26.96	0.0351	
AC	14.97	1	14.97	24.78	0.0381	
BC	0.7101	1	0.7101	1.43	0.4439	
**Residual**	1.21	2	0.6042			
**Cor Total**	175.28	7				

Table S4. Anova table for Y % resulted of Box Behnken (O-exps)

Source	Sum of squares	df	Mean square	F-value	P-value	
**Model**	659.71	9	73.30	41.51	0.0004	significant
A-Cyanex	11.42	1	11.42	6.47	0.0517	
B-Chitosan	404.85	1	404.85	229.25	< 0.0001	
C-pH	6.19	1	6.19	3.51	0.1200	
AB	0.1116	1	0.1116	0.0632	0.8115	
AC	5.12	1	5.12	2.90	0.1494	
BC	0.9643	1	0.9643	0.5461	0.4931	
A^2	73.62	1	73.62	41.68	0.0013	
B^2	34.00	1	34.00	19.25	0.0071	
C^2	153.55	1	153.55	86.95	0.0002	
ABC	0.0000	0				
**Residual**	8.83	5	1.77			
Lack of Fit	4.16	3	1.39	0.5948	0.6762	not significant
Pure Error	4.67	2	2.33			
**Cor Total**	668.54	14				

Table S5. Effect of pH of aqueous phase in the droplet size after extraction experiments

	oPE*	pH 0	pH 0.5	pH 1	pH 2	pH 3	pH 4	pH 5
**D50 (µm)**	0.83	56.14	31.44	3.73	1.04	1.10	0.96	0.90
**Mean (µm)**	0.93	52.9	29.35	4.93	1.25	1.34	1.15	1.11
**Median (µm)**	0.83	56.14	31.44	3.73	1.04	1.10	0.96	0.90

*Fresh material before contact with aqueous phase containing metals

Table S6. Equilibrium kinetics modelling parametrization

REE	Pseudo-First-Order Rate Equation (PFORE)				Pseudo-Second-Order Rate Equation (PSORE)				Elovich Equation			
	k_1 (1/min)	q_1 (mg/g)	r^2	SSE	k_2 (g/mg * min)	q_2 (mg/g)	r^2	SSE	α	β	r^2	SSE
Y	30.18	63.02	0.994	19.92	0.14	64.21	0.998	5.63	1000	0.12	0.7	921.2

Table S7. Equilibrium isotherms modelling parametrization.

REE	Langmuir				Freundlich			Sips			
	q_{max} (mg/g)	b (L/mg)	R^2	K_L at 1000 mg/L	K_F (mg/g)/(mg/L)$^{1/n}$	n	R^2	k_s (L/g)	β_s	a_s (L/mg)	R^2
Y	89.98	0.013	0.97	0.07	10.79	3.30	0.97	6.48	0.56	0.06	0.99

Table S8. Cost comparison between oPE and L-L extraction

Component	Mass/volume[1]	oPE material Price (USD)[2]	Price (USD) for treating 1 m^3 of aqueous phase
Chitosan	0.16 g	0.01584	633.6
Water	2.73 mL	9.44E-06	0.37
Acetic acid	0.028 mL	1.88E-08	0.000752
Cyanex 923	1.21 g	0.0605	2420
		Total	3053.98

Component	Mass[3]	L-L extraction Price (USD)[4]	Price (USD) for treating 1 m^3 of aqueous phase
Kerosene	22.68 g	0.013	520.00
Cyanex 923	2.32 g	0.116	4640.00
		Total	5160.00

Benchmark prices		Reference[5]
Chitosan	990 USD/10 kg	https://www.bocsci.com/
Water	3.46 USD/m^3	https://www.statista.com/statistics/1232847/tap-water-prices-in-selected-european-cities/
Acetic acid	740 USD/TM	https://www.chemanalyst.com/Pricing-data/acetic-acid-9
Cyanex 923	1000 USD/20 kg	Internal quotation (Solvay S.A.)
Kerosene	754 USD/m^3	https://www.eia.gov/dnav/pet/hist/EER_EPJK_PF4_RGC_DPGD.htm

[1] needed for making 4 g of oPE (0.14 mol/L of Cy923).
[2]for making 4 g of oPE (0.14 mol/L of Cy923).
[3]for making 25 g of Cy923/kerosene (0.25 mol/L of Cy923).
[4]for making 25 g of Cy923/kerosene (0.25 mol/L of Cy923).
[5]Data accessed: nov.10.2023.

Table S9. Parameters resulted of the evaluation of selectivity of oPE in three blends.

System	Element	Initial concentration (C_o)	Distribution coefficients (k_d)	Distribution ratio (D_{MX})	Extraction yield		Experimental sorption capacity	
		mg/L	mL/g		%	Desv	mg/g	desv
Y/Ca	Y	978.84	309.36	3.05	75.19	0.86	55.46	0.84
	Ca	405.60	13.67	0.13	11.88	0.18	3.20	0.07
Gd/Ca/Mg	Gd	2819.00	144.71	1.43	57.68	0.17	115.34	0.50
	Ca	687.08	15.48	0.15	14.10	1.74	5.59	0.76
	Mg	236.32	19.85	0.20	18.10	2.11	2.90	0.32
La/Ni	La	844.26	3405.48	33.61	97.11	0.38	53.70	0.26
	Ni	2011.31	6.75	0.07	6.24	0.12	8.23	0.08

System	Separation Factors (β1,2)
Y/Ca	22.64
Gd/Ca	9.35
Gd/Mg	7.29
La/Ni	504.64

Separation factors ($\beta_{1,2}$) were determined by Eqs. 1

$$\beta_{1,2} = \frac{D_{M1}}{D_{M2}} \tag{1}$$

where D_{M1} or D_{M2} are the distribution ratios calculated, C_0 and C_e are the initial and final REE concentration, V (mL) and m (g) are the volume and mass of oPE used in the experiments, consequently k_d is expressed in mL/g.

Table S10. Results of the application of oPE extraction experiments using an aqueous phase from fluorescent lamp powder after solid-state chlorination and leaching with HNO_3 0.01 M.

Element	C_o (mg/L) (n=3)	C_e (mg/L) (n=3)	Removal (%)	Mass transferred to oPE (mg)	q (mg/g)	Standard deviation of q
Al	15.07	15.07	0.02	0.00	0.01	0.01
Ba	717.86	669.97	6.67	47.89	4.79	1.76
Ca	20.95	20.60	1.67	0.35	0.01	0.25
Eu	71.82	52.31	27.16	19.51	1.95	0.74
Fe	5.04	3.22	36.04	1.82	0.18	0.02
Gd	0.49	0.42	13.70	0.07	0.01	0.00
Na	622.75	616.02	1.08	6.72	0.67	0.20
Nd	0.43	0.38	11.72	0.05	0.01	0.00
Y	1077.39	669.35	37.87	408.03	40.80	3.72

Table S11. X-ray fluorescence (XRF) analysis of the fluorescent lamp powder

Element	Concentration (mass %)
Si	32.91
Ba	20.57
Al	19.47
Na	5.47
Y	4.68
P	3.02
Mg	2.91
Ti	1.88
Sr	1.69
V	1.32
Ca	1.20
La	1.11
Ce	0.93
Fe	0.67
rest (concentration ≤ 0,5 mass %)	1.33

ADDITIONAL FIGURES:

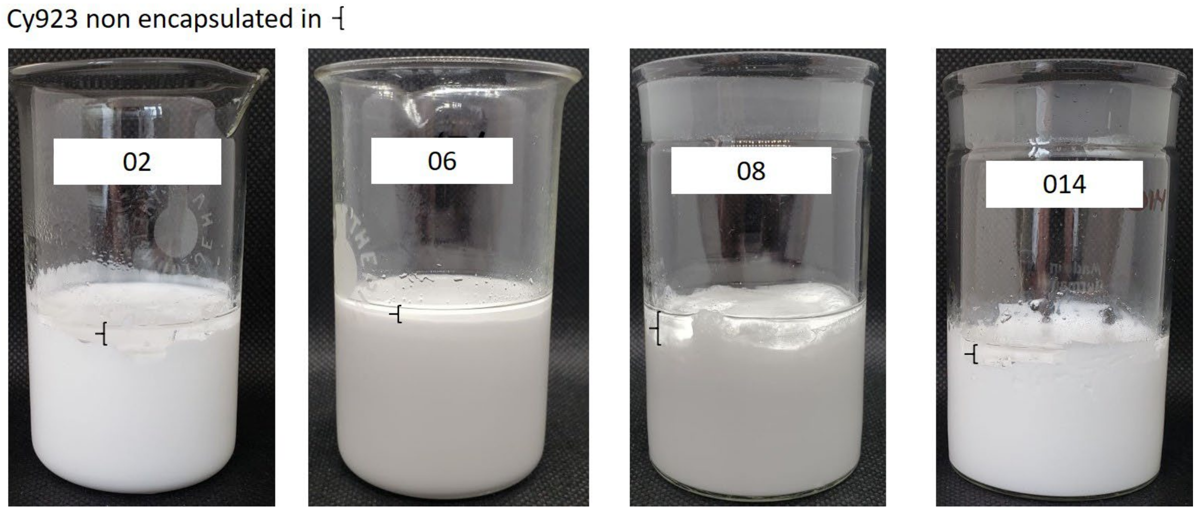

Fig. S1. Visual observations of PE experiments in where Cy923 was not encapsulated (the transparent liquid in parenthesis is Cy923).

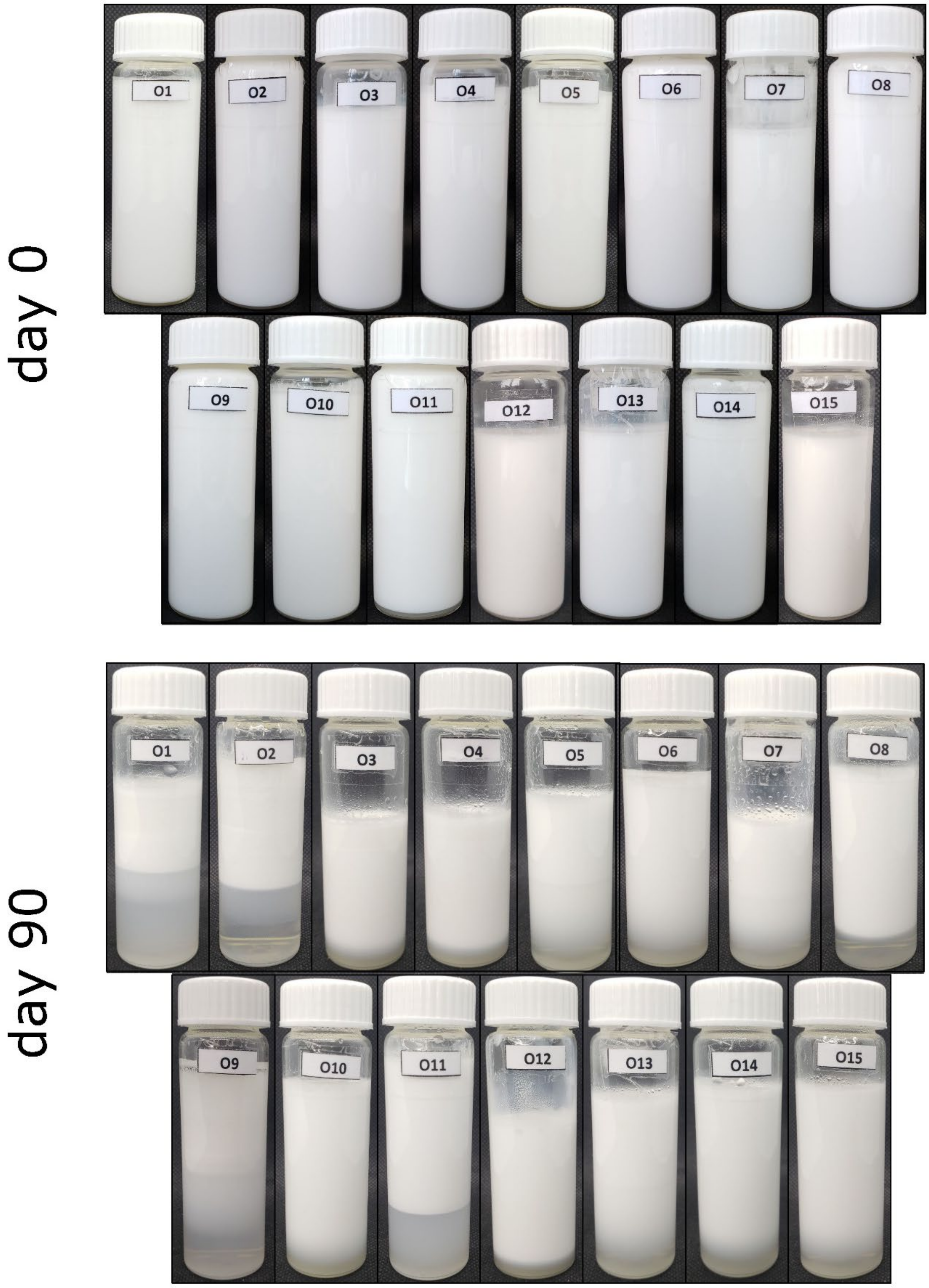

Fig. S2. Fresh and after 90 days PE appearance from O-exps

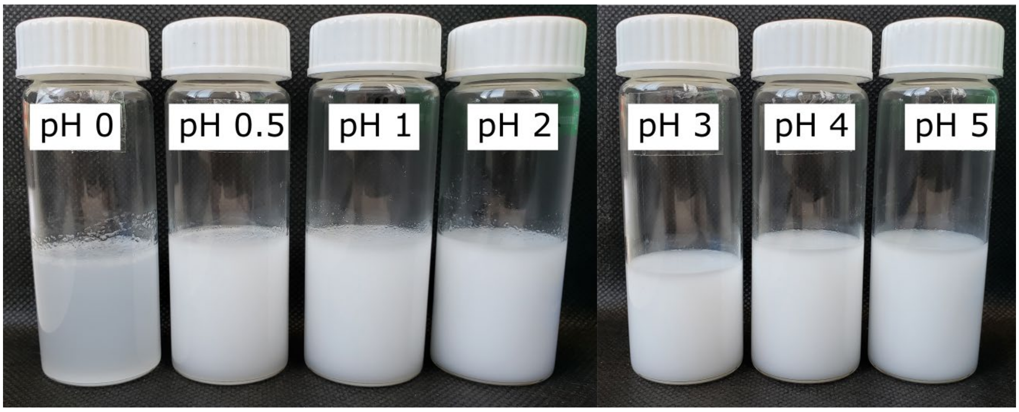

Fig. S3. Visualization of oPE extraction at different pHs

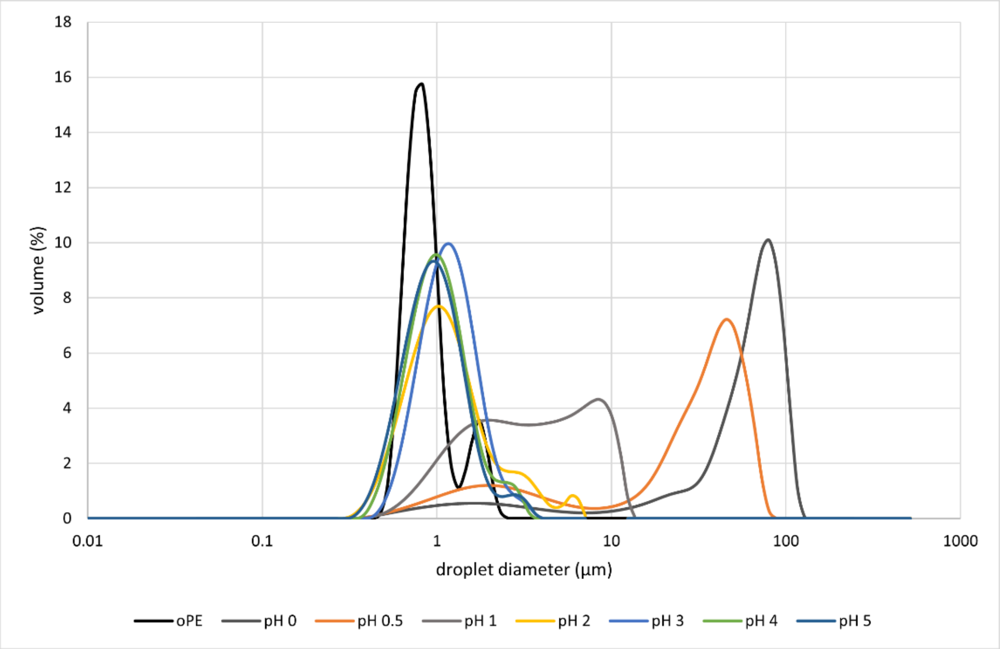

Fig. S4. Droplet size of oPE after different pHs exposition during Y extraction

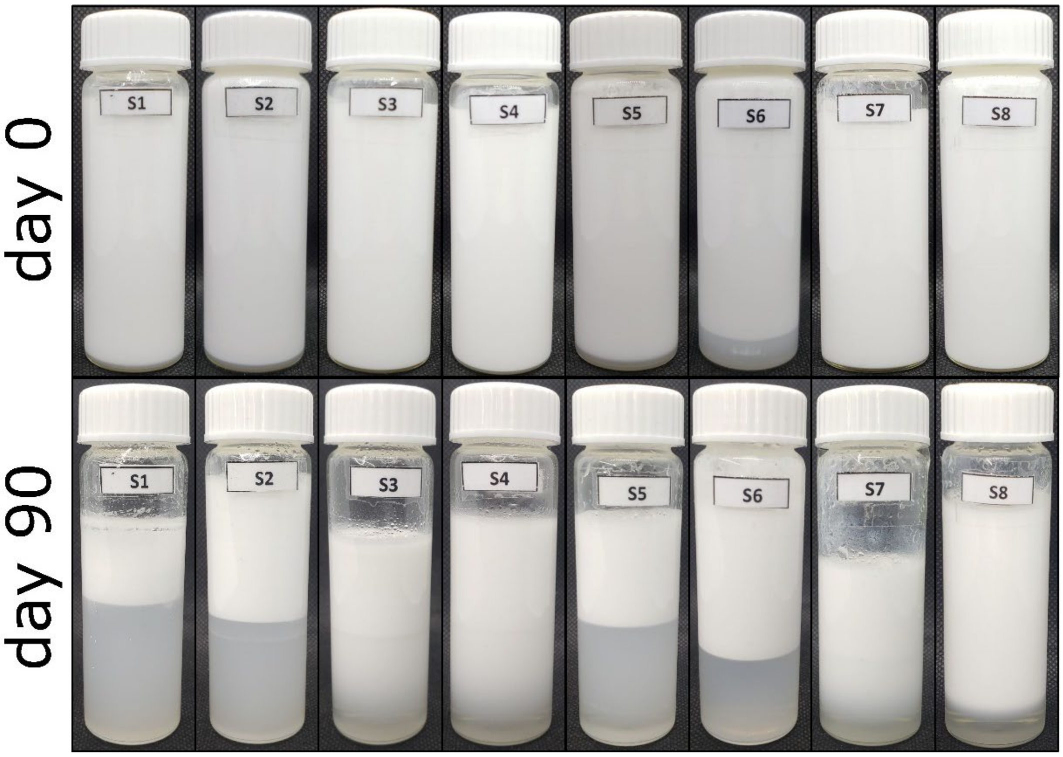

Fig. S5. Fresh and after 90 days PE appearance from P-exps
